# Contextualised reflective competence: a new learning model promoting reflective practice for clinical training

**DOI:** 10.1186/s12909-022-03112-4

**Published:** 2022-01-30

**Authors:** Andrew Stuart Lane, Chris Roberts

**Affiliations:** 1 Sydney Medical School, University of Sydney, Sydney, Australia; 2 Intensive Care Medicine, Nepean Hospital, Derby Street, Penrith, Sydney, NSW 2773 Australia; 3 Health Professions Education, and Head of Faculty Development, Sydney Medical School, University of Sydney, Sydney, Australia

## Abstract

**Background:**

Reflection is a metacognitive process that allows self-regulation and the promotion of lifelong learning, and is an essential requirement to develop therapeutic relationships with patients and colleagues, as well as professional expertise. The medical literature is lacking on guidance for learners and educators to develop reflective abilities.

**Methods:**

Based on our program of research into junior doctors delivering open disclosure communication after medical error, we developed a framework called contextualised reflective competence**,** to assist students/trainees and educators in developing, maintaining, and ensuring reflective practice in the context of professional experiences.

**Results:**

The contextualised reflective competence framework has its origins in the conscious competency framework, an established learning paradigm within healthcare professions education, and it has been developed to encompass some of the vital concepts that the conscious competency matrix was lacking: the promotion of ongoing reflection practice, accurate assumptions of the learner’s original mindset, variations in everyday performance, and erosion of skills. The contextualised reflective competence framework progresses the conscious competence framework from a 2x2 box diagram to a two-pronged flowchart.

In our framework, if the learner possesses appropriate reflective practice, contextualised reflective competence, they move through alearning process where they achieve unconscious competence. If the learner does not possess contextualised reflective competence, they move though a learning process where this display generalised reflective incompetence, characterised by cognitive dissonance and rationalisation, leading to errors and non-learning. Generalised reflective incompetence is usually a temporary state with appropriate supervision. Our program of research demonstrated that contextualised reflective competence was related to critical cognitive frameworks, such as intellectual humility, situational awareness, the development of a ‘growth mindset’, and belongingness.

**Conclusions:**

The Contextualised Reflective Competence framework promotes learners’ understanding of their core competencies and provides opportunities for personal critical reflection. It provides educators and supervisors with a diagnostic pathway for those with reflective incompetence. We anticipate its use in the clinical environment where issues of competence are raised in professional experiences.

## Background

Reflection is a metacognitive process that allows self-regulation and the promotion of lifelong learning, as well as being an essential requirement to develop both a therapeutic relationship and professional expertise [1]. The literature re-iterates the importance of reflective practice, and reflective capacity is regarded by many as an essential characteristic for professional competence [2], and has been proposed to address issues such as diagnostic decision making, medical errors, cultural competence, professionalism lapses, and burnout [3]. By being reflective, a person can ensure optimal development of their learning and understanding of their professional identity and is a recommendation and requirement of professional organisations of healthcare governance. Reflection may also help learners to integrate the emotional aspects of their learning, especially in the clinical learning environment, where students/trainees will encounter multiple challenges to their ways of thinking [2]. Therefore, students and educators need to have a greater understanding of the learning and teaching frameworks around reflective practice, to optimise learning from professional experiences [4].

There are also position statements from regulatory bodies on reflection. The American Association of Medical Colleges and Accreditation Council on Graduate Medical Education state that they expect learners and practicing physicians to use reflection to continuously improve patient care [5, 6]. In the UK, the GMC (General Medical Council) has a reflective practice statement, which outlines the principles and importance of the concept [7]. The statement outlines the expectation for clinicians to be able to synthesise and appraise their practice especially in the context of challenging periods of their practice, and consequently an ability to synthesise and appraise the context of these periods is vital. Human experiences, whether they are perceived as positive or negative, always have learning opportunities for the individuals involved. The key message of the AMMC, ACGME, and GMC statements are that reflection benefits the practitioner, colleagues, and patients, hence the importance in developing this skill.

Yet, despite reflection’s educational importance, and the presence of several helpful models in the literature, there is surprisingly little to guide educators in their work to understand and develop reflective ability in their learners [2, 8], Furthermore, models to promote reflective practice need to be grounded in areas of complex clinical practice. The emergence and development of reflective practice acknowledges the need for healthcare-professional students/trainees to act and think professionally as an integral part of learning throughout their courses of study, integrating theory and practice from the outset [2].

Recognising this gap in the literature we developed a learning and teaching framework we called contextualised reflective competence (CRC)**.** Its purpose is to assist students, trainees, and practitioners, in developing, maintaining, and ensuring reflective practice in the context of professional experiences. In this article we set out to describe the model development, and illustrate its intended use, with the expectation that it provides a useful tool to influence self-reflection.

This framework was based around our program of research into a significant issue in clinical practice, which was junior doctors delivering, and final-year medical students learning how to deliver, open disclosure communication after medication error [9]. Open disclosure is a policy stating doctors should apologise for errors, discussing them with the harmed parties. The aim of this thesis was to explore the current practice of open disclosure by junior doctors and develop an educational framework that ensured reflective clinical practice. We used the conscious competency framework of learning as part of the theoretical framework for the program of research, as this resonated with the current educational theories around how learners develop and acquire new skills, reflecting on their educational experiences as they learn [10].

We then conducted a Phenomenological study of medical interns involved in open disclosure in three parts. Firstly, ten interns were interviewed illuminating their clinical experiences of open disclosure. Eight medical students then underwent a hi-fidelity simulation session followed by focus-group discussions. Finally, the eight medical students were interviewed during their intern year, illuminating their experiences of open disclosure and their reflection on the simulation session. Data was coded and analysed using Interpretative Phenomenological Analysis (IPA). Analysis of part one demonstrated three superordinate-themes; Rationalisation of medical error; Culture of medical error; Apology in practice. Analysis of part two demonstrated two superordinate-themes; Identifying learning needs; Learning to say sorry. Analysis of part three demonstrated two superordinate-themes; Retaining learning into practice; Planning future practice. Our findings demonstrated the variety of participant experiences when engaging in open disclosure, and specifically the variety of emotions when preparing to say sorry, and after the impact of making an error. Many of these emotions resonated with aspects of the conscious competency matrix, such as feelings of conscious incompetence from the perspective of the participants, and interpretations of unconscious competence from the perspective of the researchers. Furthermore, we identified the need for educational facilitators to optimise learning for the whole group as well as the individual during simulated learning environments.

In practice the competency matrix was too simplistic for explaining the development of competency and more importantly developing a personal leaning plan for the impacted student/trainee, to address future lapses in performance. The matrix did not consider the interns’ rationalisations of why the mistake had happened, and therefore further development of the competency matrix is required to fully understand critical aspects of cognitive development for learners regarding medication error and open disclosure. Based on these findings, we revisited the current understanding of the conscious competency matrix and sought to extend it and make it adaptable to professional experiences other than open disclosure.

### Background of the conscious competence learning model

Good communication skills are vital to develop effective reflective practice. It has been well documented that a recognition of non-verbal aspects of communication, such as tone of voice and facial expressions, are more powerful when imparting communication than the actual words used themselves [11], the ability to recognise this when receiving communication is equally important. Therefore, receiving and ingesting honest and constructive feedback is as vital as delivering it, along with the ability and desire to immerse oneself on the metacognition that allows this two-way process. Reflective practice also involves an ability to recognise one’s limitations and therefore ensure subsequent life-long learning.

**Figure 1:**
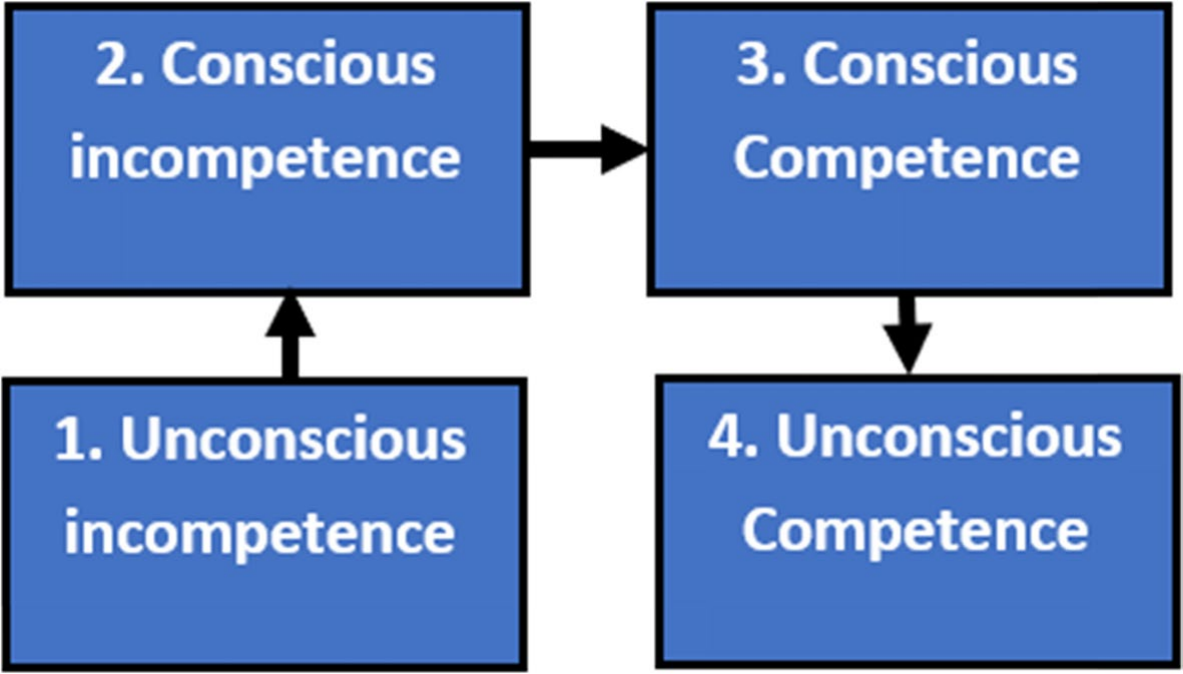
The conscious competency learning model – the four stages of learning

One of the most referenced learning frameworks in medical and healthcare profession education is the conscious competency framework. This framework was developed from the coaching industry [12–14], as a four-stage model of teaching., and the model is meant to facilitate the reflective journey in the context of developing a new skill, behaviour, ability, or technique. A commonly used version of the conscious competency learning model is outlined in Fig. 1 below:

Describing the model in more detail, learners of a new skill begin at the stage (Stage 1) of ‘unconscious incompetence’. This has been translated as somebody ‘who is not aware of what they don’t know – that they don’t realise that they cannot do the task’. As their learning of the new skills increases, they enter a stage (Stage 2) of ‘conscious incompetence’, which has been translated as ‘becoming aware they are unable to do the task. With further skill acquisition, they enter a stage (Stage 3) of ‘conscious competence’, which has been translated as ‘being aware that they are able to do the task’. Finally, as they master their skill, they attain a stage (Stage 4) of ‘unconscious competence’, which has been translated as ‘no longer being aware they can complete the task’.

There are other reflective practice cycles that are common within other professions, such as the Gibbs’s reflective cycle, leading learners through six stages of exploring an experience: description, feelings, evaluation, analysis, conclusion, and action plan. However, the conscious competency matrix is the predominant model used within the healthcare professions and clinical environments, and reflective models are universal in the principle of maintaining / progressing skills, as part of continuous professional development.

### Further development of the current conscious competency learning model

The lack of ongoing reflective practice once a skill has been learned is one of the significant flaws of the conscious competency learning model. Stage 4, also known as ‘unconscious competence’ has also been described as ‘mastery’ [15, 16] This suggests that the learner can learn no more, that they have mastered their skill. This has obvious risks in the field of medicine, where disease concepts and knowledge, investigations and management are continually changing. If a practitioner does not maintain their level of knowledge and skill, they may no longer have ‘mastery’ of their subject, which can occur if complacency enters a clinician’s practice [16]. This reinforces that idea that reflective practice is a requirement for a practitioner to maintain their mastery. However, reflective practice needs to be ongoing throughout any learning process, and subsequently throughout a practitioner’s career, always present in the background, and coming to the fore in times of need. This had led to numerous commentaries around the possibility of a fifth stage. Baume called it ‘reflective competence’, as he stated, "If unconscious competence is the top level, then how on earth can I teach things I'm unconsciously competent at?" [17] Gilbert called it ‘re-conscious competence’ indicating a stage where a person can operate with fluency on an instinctive level but are also able to articulate what they are doing for themselves and others. Dyckhoff called it ‘Chosen Conscious Competence’ and added an analogy to driving by stating ‘unconscious competence is fine when we are changing gear, but not when passing through a green light’ [17]. ‘Reflective competence’ is explained as 'conscious competence of unconscious competence', meaning a person's ability to recognise unconscious incompetence in others and themselves [18].

### Concerns regarding the conscious competency learning model

There remain significant aspects of learning that are neither demonstrated nor explained by either the original or fifth level framework. These are: whether a learner is ready to learn, can continue to learn, and is able to retain learning? In the current frameworks, learners who know they do not have a skill and are ready to learn, enter the matrix at stage 2, conscious incompetence. An example of this would be a medical student who has never performed intra-venous cannulation, who recognises that they have never done this before, understands they have no prior experience even though they might know the theory, and accepts they will make mistakes as they learn. This problem has commonly been related to and explored in early career learners [19], which can be incorrect, as they may well be more willing to accept that they have a lot to learn, compared with a mid-career learner who might believe they cannot be learn anything new.

The context of the learning must be specific to the task being performed by the learner [20]. An example of this is a junior doctor, who is learning skills within critical care medicine. They have had some previous training in Basic Life Support (BLS), and are currently consciously competent at BLS, however they are now beginning to learn bedside echocardiography of which they know they have no prior experience or training and are therefore consciously incompetent at this new skill.

The third issue is about retaining learning. Learners make mistakes as they progress from conscious incompetence to conscious competence and usually vacillate between the two stages as they learn. There also needs to be consideration for normal variations of performance, when a learner has achieved a certain level of competence, but they are having a poor performance at the time. For example, with the junior doctor learning echocardiography, they cannot recognise the parts of the ventricle that they could the previous week, as they are simply tired. The opposite end of this spectrum is when a learner is becoming overconfident. An example here using the junior doctor learning bedside echocardiography, is that they have performed 20 scans, were originally aware of their limitations and need to learn, however, they still have only rudimentary knowledge based of basic principles and are unable to interpret imaging artefacts as important structural lesions, meaning they overcall their findings. As a further extension of this, the current frameworks do not allow for preparatory reading of the procedure, and therefore learners often track the number of completed procedures as the only evidence of competency, without documented assessments of quality [21].

Finally, if reflective competence should occur at all stages of the competency matrix, a learner that possesses reflective competence should not be unconsciously incompetent. For learners who do not possess reflective competence, there needs to be an appreciation of how the thinking of the learner is accommodated within the framework, and how intervention could prevent poor reflective processes and poor learning outcomes. An example here would be where a junior doctor is assessing a patient with a headache in an emergency room, and diagnoses a migraine rather than a subarachnoid haemorrhage, as they dismiss the seriousness of the headache and the degree of hyper-reflexivity in the deep-tendon reflexes. This can occur due to cognitive bias and rationalisation. Therefore, the important aspect for learners and facilitators is not just ensuring how learners negotiate the competency framework, but how they are cognitively situated before they commence the learning process, and how they will remain cognitively situated throughout their ongoing learning.

## Methods

**Figure 2:**
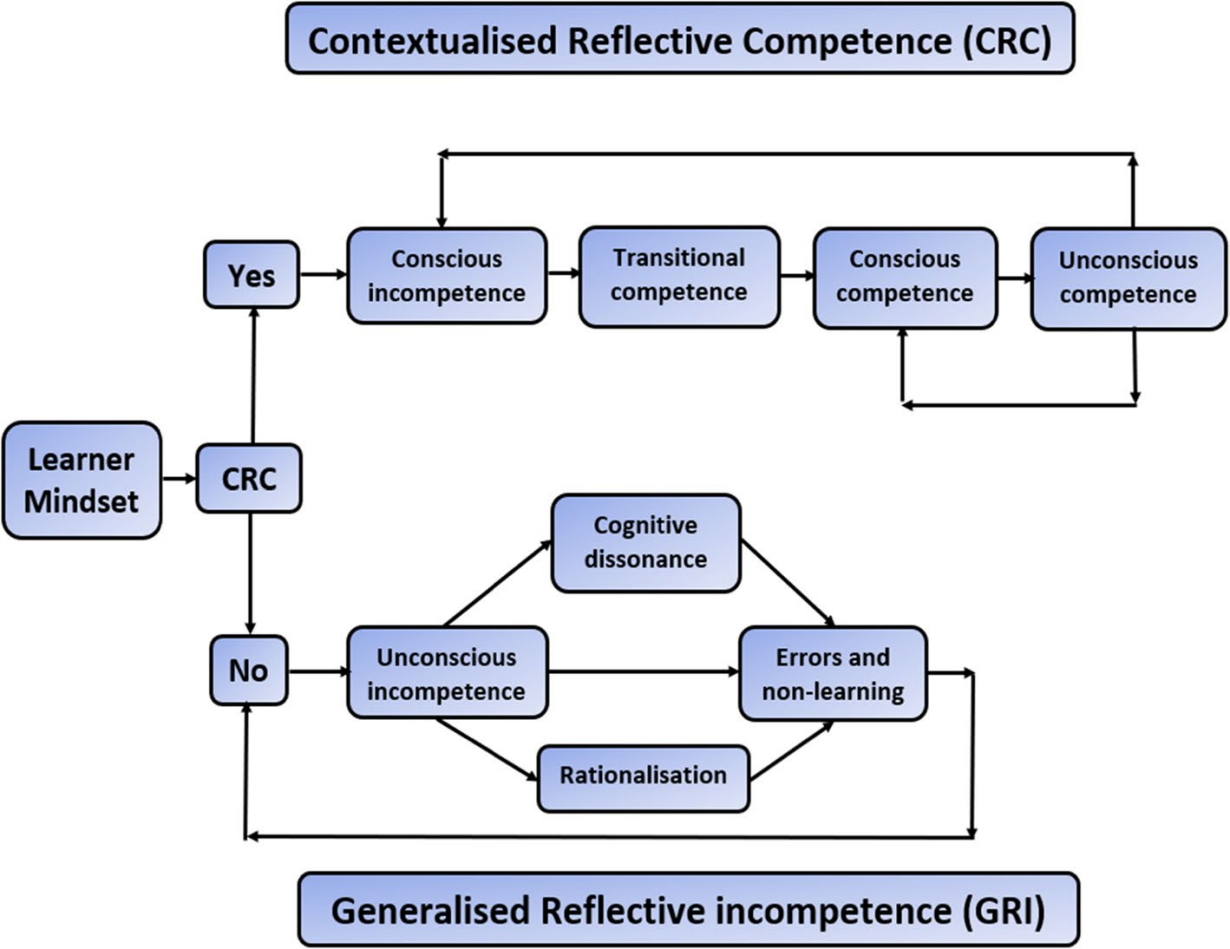
Learning model for ‘contextualised reflective competence’

Based on our program of research of junior doctors’ experiences of open disclosure after medication error [9] as well final-year medical students learning of open disclosure via simulation [10] and using the conscious competency framework of learning as a conceptual framework to illuminate our research findings, we propose the contextualised reflective competence framework (CRC). Demonstrated in Fig. 2, we believe that the CRC framework resonated with the interns’ rationalisation of the difficulties in their clinical practice and the clinical environment around them.

## Results

In this generalised mode, we assume the learner experiences a critical incident or encounters a new learning paradigm, for example making a prescribing error, demonstrating a lapse of professionalism, or having situational awareness of their inability to contribute to care, for example in advanced life support. If the learner possesses the appropriate reflective practice, as in they possess CRC, then they move though the upper loop of the framework, moving towards and achieving unconscious competence. If the learner does not possess the appropriate reflective practice, as in they do not possess CRC, then they move though the lower loop of the framework, and their subsequent learning and behaviours are described around generalised reflective incompetence (GRI). This GRI is envisaged as a temporary state where there is appropriate supervision or mentorship, and in the absence of these it may be a prolonged state. We will now consider the learning consequences of both pathways in more detail.

### Reconceptualising the conscious competency learning model: Contextualised reflective competence

The learner embarks on the learning of a new task, possesses CRC, and moves via the ‘YES’ box to the stage of being consciously incompetent. They know they that cannot do a task, with an acceptance that they may make mistakes and learn from them. As the intern progresses through the various stages of competency, they go through learning stage where they are aware, they are getting it right sometimes, and wrong at other times. This is a new middle stage in diagram, reflecting a transitional learning stage which we have labelled transitional competence. As they develop their skills further, they become aware they are consistently getting it right, and they are now at the stage of conscious competence. As they progress further with the learned skill, they progress to unconscious competence. This model also allows for the development of overconfidence during the transitional competence stags of learning, since if this occurs, they no longer possess CRC, and they will then move to the bottom level of the learning model where they will make errors and enter a cycle of non-learning.

The upper part of the diagram is described as having the reflective ability of ‘Context Reflective Competence’ (CRC), which can be practically explained as reflecting with ‘the right people, at the right time, in the right manner’. Because they have CRC for that specific task, they remain on the upper part of the diagram, whatever their level of attained competency or actual daily performance. This context is important as it allows for episodes of poor performance and the decaying of skills, since the practitioner can recognise that they are once again aware of the task they are doing, either correctly or incorrectly, when they previous had not been aware due to being at the level of unconscious competence. This connection is demonstrated by the arrows on the diagram going back from unconscious competence to conscious incompetence and conscious competence, and as they are conscious of their functioning, they remain on the top part of the diagram.

### Reconceptualising the conscious competency learning model: Generalised reflective incompetence

The learner embarks on the learning of a new task, does not possess CRC, and moves via the ‘NO’ box to the stage of being unconsciously incompetent. They have no awareness that they cannot perform a task, and do not recognise the errors that they are making. There are three potential pathways that come out of the unconscious incompetence box on the diagram, with arrows that go to ‘errors and non-learning’ via cognitive dissonance, rationalisation, or neither. The bottom part of the diagram is described as having a mindset of ‘Generalised reflective incompetence’ – which can be practically explained as reflecting with the wrong people, at the wrong time, in the wrong manner.

Looking at the three potential pathways on the lower part of the diagram in more detail, the pathways reflect three different cognitive processes that all eventuate in errors and non-learning. Cognitive dissonance is the feeling of discomfort when simultaneously holding two or more conflicting cognitions: ideas, beliefs, values, or emotional reactions [22]. This contradiction between two beliefs will spontaneously create a third belief in order tom reduce the dissonance. Generally, this 'third belief' is pure confabulation [23]. Cognitive dissonance is a largely unconscious process; you are seldom consciously aware that you hold two contradictory beliefs or value systems simultaneously, utilising each belief only when it is most socially convenient to do so. Many of the junior doctors in our research spoke of an uncomfortable feeling when they were giving open disclosure, a feeling that something was not quite right in their practice and they were not quite sure what it was, yet they persisted with their chosen approach without further clarification. One of the outcomes of cognitive dissonance is a rationalisation, where the person makes a justification of their actions [24]. The process of rationalisation or ‘blame-shifting’ is a defence mechanism in which controversial behaviours or feelings are justified and explained in a seemingly rational or logical manner, to avoid the true explanation, and are made consciously tolerable—or even admirable and superior—by plausible means [25]. Rationalisation is common when doctors make an error, with the objective of rationalisation being for the professionals to convince themselves that error concealment (or a dissimulating disclosure) is not morally or professionally wrong [25]. The rationaliser wants to be convinced of the rightness of his or her concealment or dissimulation, because moral people do not violate ethical obligations connected with veracity, honesty and other moral norms associated with the fiduciary nature of the professional-patient relationship.

Tsang describes ways in which rationalisation, the process of re-interpreting the moral situation is achieved [26]. Examples of these re-interpretations within Medicine are: the use of euphemistic language - choosing words and phrases to conceal harm, such as calling the outcome of an error a ‘complication’, advantageous comparison - comparing the concealment to something worse, such as believing that telling the patient will only make the patient feel worse, and distorting the consequences of an action - reinterpreting the consequences in a positive light, such as saying that a lethal error was a ‘blessing in disguise’ or a ‘learning experience’ [26]. These findings of rationalisation were also consistent with our own research, one the example being an intern who was apologising for a patient receiving too much anticoagulation for a deep vein thrombosis which they believed that this was a good thing as the patient was now ‘super-anticoagulated’. This example displays both the use of euphemistic language and advantageous comparison.

Finally, the person may neither experience cognitive dissonance, nor perform rationalisation, and simply have no awareness of their mistakes. All these pathways involve poor reflective practice, and all demonstrate the learner is unconsciously incompetent.

Generalised reflective incompetence should be a temporary state for most leaners, since if they have the appropriate supervision and receive timely and constructive feedback, they will be able to reflect appropriately and regulate their learning, and hopefully have better self-regulation in the future. Therefore, as educators, if we are to prevent learners becoming stuck in the endless loop of generalised reflective incompetence, we need to be able to recognise these cognitive frames in learners, and if they are not able recognise these frames themselves, be prepared initiate the conversations that can lead to remodelling of these frames.

### Reconceptualising the conscious competency learning model: Further discussions of CRC

Whilst ‘reflecting with the right people, at the right time, in the right manner’ is a practical way to describe CRC, the construct itself is difficult and complex to define and is made up of numerous components. Our research demonstrated that it is related to certain cognitive frameworks which were identified in our data. Examples of cognitive frameworks that might influence the ability to possess CRC were;

- intellectual humility [27, 28] - having a consciousness of the limits of one's knowledge, including a sensitivity to bias, prejudice, and limitations of one's viewpoint. Intellectual humility depends on recognising that one should not claim more than one knows.
- situational awareness [29] - ‘the perception of elements in the environment within a volume of time and space, the comprehension of their meaning, and the projection of their status in the near future’, People with good situational awareness have a good ‘feel’ for situations and people, and events that play out due to variables the subject can control.
- the development of a ‘growth mindset’ [30] – where individuals do not mind or fear failure as much because they realise their performance can be improved and learning comes from failure, and therefore it is possible to encourage students to persist despite failure by encouraging them to think about learning in a certain way
- belongingness [31, 32] - the human emotional need to be an accepted member of a group, and the development of professional identity [33, 34], and the meanings that individuals hold for themselves, what it means to be who they are’

The previous literature refers to unconscious competence being a state of ‘mastery’, however we have omitted this term from the framework. Even when a learner has progressed to the level of unconscious incompetence, as described previously, they can still have episodes of poor performance or start to have a decaying of skills but remain on the upper level of the diagram since they recognise the lapse. The importance of mastery is not about the level of attainment of a new skill, it is the ability to reflect appropriately at every point of learning and performance. Mastery is possessing contextualised reflective competence.

The data from our studies and this newly developed framework demonstrate the need for appropriate personal critical reflection, mentorship, and guidance, to ensure that learners stay on the correct level of the framework (the upper level). Since mentors and educators are not always physically present to assist a learner’s development, it is therefore imperative that they impart the ability and the desire for the learner to develop and continue to possess contextualised reflective competence (CRC). This means educators must ensure learners develop the correct mindset before they commence learning and instil a desire and ability to maintain or regain the correct learning mindset throughout their future career. Thus, the learner remains on the upper part of Fig. 2 and sustaining contextualised reflective competence (CRC).

## Conclusion

We describe a learning model we have called Contextualised reflective competence**.** Its purpose is to assist students, trainees, and practitioners in ensuring reflective practice in the context of professional experiences. It promotes learners’ understanding of their core competencies and provides opportunities for critical reflection of their own efforts encouraging them to become more autonomous learners. It provides educators and supervisors with a diagnostic pathway for those with reflective incompetence. We anticipate its use in the clinical environment where issues of competence are raised in professional experiences.

## Data Availability

The datasets generated and/or analysed during the current study are not publicly available due to not being stored on a publicly accessible platform but are available from the corresponding author on reasonable request.

## References

[1] Sandars J (2009). The use of reflection in medical education: AMEE Guide No. 44. Medical Teach.

[2] Mann K, Gordon J, MacLeod A (2009). Reflection, and reflective practice in health professions education: a systematic review. Adv Health Sci Education.

[3] Hodges BD (2015). Sea monsters & whirlpools: Navigating between examination and reflection in medical education. Med Teach..

[4] Aronson L (2011). Twelve tips for teaching reflection at all levels of medical education. Medical teacher..

[5] Swing SR (2007). The ACGME outcome project: Retrospective and prospective. Med Teach..

[6] Association of American Medical Colleges. Core Entrustable Professional Activities for Entering Residency: Curriculum Developer’s Guide 2014. Washington, DC: Association of American Medical Colleges; https://members.aamc.org/eweb/upload/Core%20EPA%20Curriculum%20Dev%20Guide.pdf

[7] The reflective practitioner – guidance for doctors and medical students. https://www.gmc-uk.org/education/standards-guidance-and-curricula/guidance/reflective-practice/the-reflective-practitioner%2D%2D-guidance-for-doctors-and-medical-students

[8] Nguyen QD, Fernandez N, Karsenti T, Charlin B (2014). What is reflection? A conceptual analysis of major definitions and a proposal of a five-component model. Medical education..

[9] Lane AS, Roberts C (2020). Rationalisation of medication error by medical interns - linking the competency matrix to professional learning and reflective practice.

[10] Lane AS, Roberts C. Developing open disclosure strategies to medical error, using high-fidelity simulation in medical school. 2021. BMJ Simulation Technology and enhanced learning https://stel.bmj.com/content/early/2020/12/02/bmjstel-2020-000659 10.1136/bmjstel-2020-000659 PMC8936528 35515741

[11] Mehrabian A. Non-verbal communication. 1972. Chicago IL Aldine-Atherton

[12] Broadwell MM. Teaching for learning (XVI). The Gospel Guardian. 20 February 1969.

[13] Curtiss PR, Warren PW (1973). The dynamics of life skills coaching. Life skills series. Prince Albert, Saskatchewan: Training Research and Development Station, Dept. of Manpower, and Immigration.

[14] Conscious competence learning model. four stages of learning theory - unconscious incompetence to unconscious competence matrix - and other theories and models for learning and change. Available at: http://www.psychology-solution.com/confidence/competence last accessed??

[15] Van der Vleuten C, Schuwirth L, Driessen E, Dijkstra J (2012). A model for programmatic assessment fit for purpose. Medical Teacher.

[16] Barrett L (1999). The Structure of Current Affect: Controversies and Emerging Consensus. Current Directions in Psychological Science..

[17] Leadership and management training. Fifth level of reflective competence. [cited 2020 Jun 21]. Available at: https://www.businessballs.com/self-awareness/conscious-competence-learning-model/

[18] Dekker H, Schonrock-Adema J. Snoek JW, Van der Molen T, Cohen-Schotanus J. Which characteristics of written feedback are perceived as stimulating students' reflective competence: an exploratory study. 2013. BMC Medical Education. Vol 13, Article 94. 10.1186/1472-6920-13-94 PMC3750500 23829790

[19] Van den Eertwegh V, Van der Vleuten C, Stalmeijer R, Van Dalen J, et al. Exploring Residents’ Communication Learning Process in the Workplace: A Five-Phase Model. PLoS One. 2015;10(5). 10.1371/journal.pone.0125958 PMC4441458 26000767

[20] Lane AS, Orde SR. Intermediate level training: a paradigm requiring reflective competence. Journal of the Intensive Care Society. Volume 18, Issue 1, February 2017 76-80 52-56 10.1177/1751143716664107 PMC5606358 28979545

[21] Manthey D, Fitch M (2012). Stages of competency for medical procedures. The clinical teacher..

[22] Oxford Dictionaries. Language matters. [cited 2020 Jun 21]. Available at: http://www.oxforddictionaries.com/definition/english/light-bulb-moment

[23] Herstein W. Brain Fiction: Self-deception and the Riddle of Confabulation. The MIT press. 2005.

[24] Reason J (1990). Human error.

[25] Banja J. Medical Errors and Medical Narcissism. Jones and Bartlett. 2005.

[26] Tsang J. Moral rationalisation and the integration of situational factors and psychological processes in immoral behaviour. Review of general psychology. 2002. 6(1) p25-50.

[27] Valuable intellectual traits. The foundation for critical thinking. [cited 2020 Jun 21]. Available at: http://www.criticalthinking.org/pages/valuable-intellectual-traits

[28] The theology and philosophy of intellectual humility. John Templeton Foundation. [cited 2020 Jun 21]. Available at: https://www.templeton.org/what-we-fund/grants/the-philosophy-and-theology-of-intellectual-humility

[29] Endsley M (1995). Toward a theory of situation awareness in dynamic systems. Human Factors..

[30] Dweck CS. Mindset: How You Can Fulfil Your Potential. Constable & Robinson Limited. 2012.

[31] Fiske ST (2004). Social beings: A core motives approach to social psychology.

[32] Zimmerman BJ (2002). Achieving self-regulation: The trial and triumph of adolescence. Academic motivation of adolescents.

[33] Burke P. Identities and Social Structure: The 2003 Cooley-Mead Award Address Social Psychology Quarterly March 2004. Vol. 67 no. 1 5-15

[34] Tajfel H, Turner J (1986). The Social Identity Theory of Intergroup Behaviour.

